# A Polyphenols-Rich Extract from *Moricandia sinaica* Boiss. Exhibits Analgesic, Anti-Inflammatory and Antipyretic Activities In Vivo

**DOI:** 10.3390/molecules25215049

**Published:** 2020-10-30

**Authors:** Sahar El-mekkawy, Abdelaaty A. Shahat, Ali S. Alqahtani, Mansour S. Alsaid, Mohamed A.O. Abdelfattah, Riaz Ullah, Mahmoud Emam, Abdelaziz Yasri, Mansour Sobeh

**Affiliations:** 1 Department of Chemistry of Natural Compounds, National Research Centre, Dokki, Giza 12622, Egypt; saheg.2011@gmail.com; 2 Pharmacognosy Department, Medicinal Aromatic and Poisonous Plants Research Center, College of Pharmacy King Saud University, Riyadh 11451, Saudi Arabia; alalqahtani@ksu.edu.sa (A.S.A.); msalsaid@ksu.edu.sa (M.S.A.); rullah@ksu.edu.sa (R.U.); 3 Chemistry of Medicinal Plants Department, National Research Centre, Dokki, Giza 12622, Egypt; 4 College of Engineering and Technology, American University of the Middle East, Kuwait; mohamed.abdelmoety@aum.edu.kw; 5 College of Pharmaceutical Science & Collaborative Innovation Center of Yangtze River Delta Region Green Pharmaceuticals, Zhejiang University of Technology, Hangzhou 310014, China; 6 Phytochemistry and Plant Systematics Department, National Research Centre, Dokki, Giza 12622, Egypt; 7 AgroBioSciences Research Division, Mohammed VI Polytechnic University, Lot 660–Hay MoulayRachid, 43150 Ben-Guerir, Morocco; aziz.yasri@um6p.ma

**Keywords:** *Moricandia sinaica*, analgesic, anti-inflammatory, antipyretic, LC-MS/MS

## Abstract

In this study, the aerial parts of *Moricandia sinaica* were evaluated for their in vivo analgesic, anti-inflammatory and antipyretic activities. The analgesic activities were examined using acetic acid-induced writhing, the hot plate test and the tail flick method. The anti-inflammatory and the antipyretic activities were evaluated using carrageenan-induced paw edema in rats and brewer’s yeast-induced pyrexia in mice, respectively. The aqueous fraction of the methanol extract (MS-3) showed to be the most bioactive among the other investigated fractions. At the dose of 500 mg/kg, the fraction (MS-3) showed a significant percentage inhibition of the carrageenan-induced edema by 52.4% (*p* < 0.05). In addition, MS-3 exhibited a significant inhibition of acetic acid-induced writhes by 44.4% and 61.5% (*p* < 0.001) at 250-mg/kg and 500-mg/kg doses, respectively. At 120 min post-treatment, the rat groups treated with MS-3 displayed statistically significant reduction in rectal temperature (*p* < 0.001) by 1.7 °C and 2.2 °C at 250- and 500-mg/kg doses, respectively. The phytochemical composition of the fraction (MS-3) was characterized by high-performance liquid chromatography-mass spectrometry (HPLC-PDA-MS/MS). Molecular docking studies demonstrated that the polyphenols identified in MS-3 revealed good binding energy upon docking to some target proteins involved in pain response and inflammation, such as the cannabinoid receptors CB1 and CB2, the fatty acid amide hydrolase (FAAH) and the cyclooxygenase COX-1 and COX-2 enzymes. Based on the findings from the present work, it could be concluded that the aerial parts extract of *M. sinaica* exerts potential analgesic, anti-inflammatory and antipyretic effects in rats.

## 1. Introduction

Inflammation is the body’s protective immune response that guards against cell injury and initiates tissue repair. It is triggered by a variety of stimuli, such as microbial invasion, tissue damage, ultraviolet irradiation and exposure to chemical irritants, among others. Failure to initiate an inflammatory response could compromise the endurance of the invasive organism or lead to tissue destruction by the deleterious stimulus [1]. However, chronic inflammation is linked to the development of several pathological conditions, such as rheumatoid arthritis, asthma, psoriasis, multiple sclerosis and inflammatory bowel disease. Such conditions are debilitating and getting more common within the aging society. Currently used anti-inflammatory drugs suffer either numerous side effects or a relatively higher cost of treatment. Alternatively, natural plants and their derived isolated compounds offer a great option to identify novel bioactive leads for the development of cost-effective and powerful anti-inflammatory candidates with lower side effects [1,2,3].

The mustard family, Brassicaceae, represents a vital part of the human diet all over the world. It comprises about 338 genera and 3709 species, with high economic importance owing to various species producing food and oilseed crops, as well as other several species of ornamental plants and noxious weeds [4]. Plants of the genus *Moricandia* have shown some potential health benefits [5]. For instance, the leaves of *M. arvensis*, which serve as a common ingredient in traditional cooking recipes in Tunisia, are used in the treatment of syphilis [6]. Moreover, some extracts from the plant were reported to exhibit antioxidant and antigenotoxic properties and effectively reduced the proliferation of human cancer cells [7,8,9].

*Moricandia sinaica* Boiss. is an annual-to-perennial suffrutescent-to-suffruticose plant, which is native to the Mediterranean region, Europe and America. However, the plant species are cultivated presently worldwide as ornamental plants with violet, purple and white flowers [10]. To date, there are no available studies reporting the pharmacological activities or the phytochemical characterization of *M. sinaica*. In this study, the analgesic, anti-inflammatory and antipyretic activities of *M. sinaica* extracts and fractions were evaluated in vivo. The phytochemical profile of the most bioactive fraction was characterized by liquid chromatography-mass spectrometry (LC-MS/MS), ^1^H-NMR data and a molecular networking analysis. Molecular docking studies of the identified compounds to some target proteins involved in pain response and inflammation were carried out.

## 2. Results and Discussion

### 2.1. Phytochemical Analysis

The exposure of the *M. sinaica* fraction (MS-3) to ferric chloride (1%) led to an intense green color, which might be attributed to the presence of dihydroxy phenolics. In Shinoda’s test, a positive red color was remarkable due to the presence of flavonoids and/or their glycosides. While the light orange (red) color was attributed to the traces of nitrogenous compounds in Dragendroff’s test. On the other hand, fatty acids, terpenes and saponins constituents were absent.

**Figure 1:**
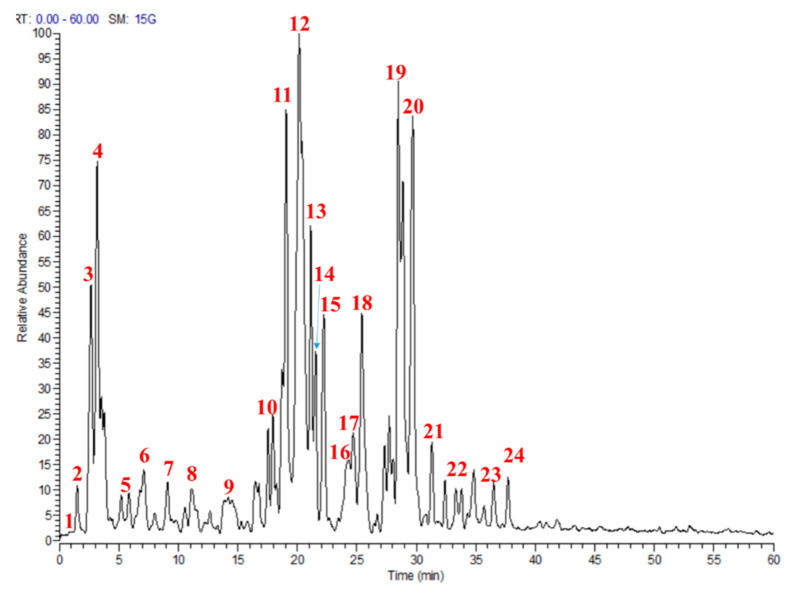
Profile of the active fraction from *Moricandia sinaica* aerial parts (MS-3) using liquid chromatography-mass spectrometry (LC-MS). Numbers in the figure match numbers of secondary metabolites in Table 1.

Utilizing a bio-guided assay, the most bioactive fraction from *M. sinaica* (MS-3) was identified, and the secondary metabolites were characterized based on their molecular weights, mass fragmentation and retention times using LC-MS/MS. Altogether, 24 compounds were annotated; among them, flavonoid glucosides (isorhamnetin, quercetin and kaempferol) dominated the fraction (Table 1 and Figure 1).

#### 2.1.1. Molecular Networking of *M. sinaica* Aerial Part (MS-3) Metabolites

**Figure 2:**
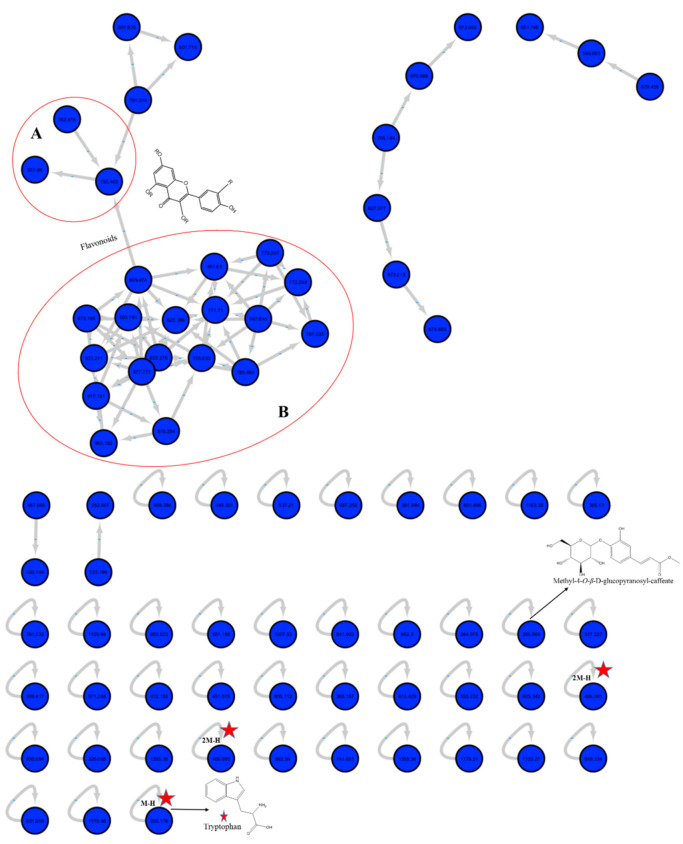
Full molecular networking from *M. sinaica* aerial parts fraction created using MS/MS data in negative mode. Nodes are labeled with parent mass. Clusters A and B indicate the *O*-glycosidic flavonols and their derivatives.

The molecular networking is based on the similarity of definite mass fragments for similar metabolites that facilitate the visualization of identical chemical entities. At the network, each node was labeled with the precursor mass, and nodes having identical fragments were connected with edges (Arrows). Each edge has the mass loss on its arrow. The network was constructed for the negative ionization mode using the GNPS 2 platform (Figure 2). The network contained 78 nodes and 41 self-looped nodes. The designed networks facilitated the visual examination of the different compound families, analogs and assisted in isomers differentiation.

In the negative network, clusters A and B implicated the *O*-glycosidic flavonols and their derivatives. Some other self-looped nodes were identified within the network and represented as phenolic-*O*-glycosides, alkaloids, phenolic acids and their derivatives.

#### 2.1.2. LC-MS/MS Metabolites Explanation

Different organic acids and their derivatives, glucosinolate and flavonoids were identified as the following; Acids and their derivatives: peak 1 appeared at a retention time (t_R_) of 0.92 min; showed an ([M − H)^−^ ion at *m/z* 317 and produced fragment ions 225, 165 and 125 in MS^2^ spectra that are characteristics for galloyl phloretic acid. The (M-H)^-^ ion at *m/z* 341 yielded a molecular ion peak at *m/z* 179 (M – H − 162)^−^ corresponding to the loss of the glucosyl moiety; it was annotated as caffeoyl glucose [11]. Peak 4 retained at a t_R_ of 3.53 min; gave an (M – H)^−^ ion at *m/z* 133 and exhibited the significant fragment ions *m/z* 114, 115, 87 and 71 in MS^2^ that are characteristic for malic acid [12]. Peak 5 (*m/z* 385) suggested the occurrence of a sinapoyl hexoside structure, which was confirmed by the presence of fragment ions at *m/z* 223 (sinapic acid – H)^−^ (after losing the hexosyl moiety), *m/z* 205 (sinapic acid – H − H_2_O)^−^ (after losing the water molecule) and *m/z* 179 (sinapic acid − H − CO_2_)^−^ (after losing the CO_2_ molecule) [13]. In addition, the retained peak at 7.11 min with an (M − H)^−^ ion at *m/z* 163 yielded significant product ions *m/z* 147, 128 and 119; it was characterized as *p*-coumaric acid [14]. Peak 8 was eluted at 11.12 min; had a (M-H)^-^ ion at *m/z* 355 and fragment ions at *m/z* 338, 309 and 193 in MS^2^ chromatogram that could be characterized as methyl-4-*O-β*-D-glucopyranosyl-caffeate [15]. Glucosinolate: peak 3 was observed at **a** t_R_ of 3.17 min; showed an (M − H)^−^ ion at *m/z* 372 and gave quasi-fragment ions for glucosinolates at *m/z* 292, 276, 259, 241, 209, 194 and 178 that could be justified by the loss of SO_3_H, SO_4_H, SO_3_H + H_2_O, SO_3_H + 2H_2_O and glucosyl moieties, respectively, and, thus, could be identified as gluconapin [16].

Flavonoid glycosides: flavonoid –*O*-di-, tri- and/or tetra-glycosides were tentatively identified at a t_R_ of 17 to 37.7 min (peaks 10–24). Quercetin, kaempferol and isorhamnetin were confirmed by the characteristic fragment ions at *m/z* 301, 285 and 315 for different aglycones, respectively. The characteristic fragment ions observed after the loss of 162 and 146 Da are indicative of hexoses and rhamnoses moieties, respectively. The loss of the feruloyl structure was confirmed by the loss of 176 Da [17]. Alkaloid: tryptophan was tentatively identified depending on the GNPS libraries, which showed (M − H)^−^ and (2M − H)^−^ ions at *m/z* 203 and 407, respectively.

#### 2.1.3. ^1^H-NMR Analysis of the *M. sinaica* Fraction

The aqueous fraction was dissolved at DMSO-*d6* and introduced to the ^1^H-NMR (500 MHz) experiment. The observed peaks were resonated, along with different chemical shifts at different regions. The *Sp*^2^ aromatic region exhibited different splitting numbers of signals at δ 8.04 (br s), 7.76 (br s), 7.66 (d), 7.39 (d), 7.25 (d), 7.20 (m), 7.12 (m), 7.03 (d), 6.85 (d), 6.66 (d), 6.42 (d) and 6.15 (d) that explained the *m-* (*J* = 2–3 Hz) and *o-* (*J* = 7–8 Hz) coupling of different aromatic protons of flavonoids (B-ring protons). The downfield shift of δ 6.66 and 6.4 may be attributed to the presence of substitutions at position 7 of the flavanols. In addition, the chemical shifts that appeared at 5.22 (d), 5.17(d), 4.41 (d) and 4.31 (d) are characteristic for the anomeric protons of the different sugar moieties substituted at the 3 and 7 positions of the flavonoids; one of them is rhamnose, which is characterized by the methyl doublet signal at δ 0.91 with a coupling constant *J* = 6.2 Hz, and the other is glucoside, which is explained by the coupling constant *J* = 7.5 Hz at δ 5.27. The downfield shift of the rhamnose anomeric proton to δ 5.17 is attributed to the rhamnose that is directly attached to position 3 or 7 of the flavonols. The different chemical shifts of the rest of the sugar protons resonated around δ of 3.76 to 1.76. Finally, the methoxy group of the isorhamnetin derivatives was characterized at δ of 3.88.

### 2.2. Total Phenolic Content (TPC) and Total Flavonoid Content (TFC)

The Brassicaceae family is a rich source of natural polyphenols with considerably diverse health-promoting and antioxidant properties [18]. The bioactive fraction (MS-3) exhibited solid antioxidant activities in vitro, where it demonstrated an EC_50_ of 12.31 ± 0.15 μg/mL in the DPPH assay. In addition, it showed a high total phenolic (TPC) and flavonoid (TFC) contents, found to be 203.73 ± 0.24 μg-GAE/mg and 114.09 ± 0.04 μg-QE/mg, respectively. Similar antioxidant properties and phenolic contents were reported for the leaves and aerial parts from *M. arvensis* [18].

### 2.3. Analgesic Effects of M. sinaica Extract and Fractions

The analgesic activities of *M. sinaica* fractions were evaluated by three different methods, namely the hot plate test, acetic acid-induced writhing and the tail flick method. Regarding the hot plate test, the activities of the three fractions are summarized in (Table 2). In particular, the *M. sinaica* fraction (MS-3) exerted an appreciable effect in the high-dose level (500 mg/kg), where it prolonged the reaction time significantly (*** *p* < 0.001) by 10.5, 11.83 and 11.66 min after 30, 60 and 90 min of the fraction administrations, respectively. This effect is moderately potent when compared to the positive control indomethacin.

In the acetic acid-induced writhing model in mice, *M. sinaica* fractions decreased significantly (*p* < 0.001) the number of acetic acid-induced writhes counted within 20 min and showed a moderately potent analgesic effect when compared to the positive control indomethacin. The fraction MS-2, at a 500-mg/kg dose, showed a significant inhibition of writhes by 41.2% (*p* < 0.001), while the MS-3 fraction showed significant inhibitions of 44.4% and 61.5% (*p* < 0.001) at 250-mg/kg and 500-mg/kg doses, respectively (Table 3).

The tail flick method was utilized to confirm the analgesic activities of the fraction by focusing an intensity-controlled beam of light on the animal tail. The reaction time was measured at 60, 90 and 120 min after the administration of 250 and 500 mg/kg of *M. sinaica* fractions. All fractions prolonged the response time duration in this bioassay (Table 4).

However, the most effective fraction was MS-3 at the dose 500 mg/kg, in which the response time duration was prolonged to 7.83 min, 9.16 min and 8.66 min after 30, 60 and 120 min post-treatment, respectively, compared to the 5.16-min response time duration showed by the control group (without treatment).

### 2.4. Anti-Inflammatory Activity in Carrageenan-Induced Paw Edema

In another approach, we evaluated the anti-inflammatory activities of *M. sinaica* fractions. They showed moderate potency to inhibit the carrageenan-induced edema in the rats’ paw model. At the low-dose level (250 mg/kg), MS-3 and MS-2 fractions of *M. sinaica* showed 45% and 31.3% inhibition, respectively, which is nearly half the potency exhibited by the positive control oxyphenbutazone. At the high-dose level (500 mg/kg), both fractions showed a significant percentage inhibition of edema formation by 52.4 and 50.4, respectively (*p* < 0.05) (Table 5).

### 2.5. Antipyretic Activity in Yeast-Induced Hyperthermia in Mice

To run a comprehensive approach, we explored the antipyretic activity of the different *M. sinaica* fractions. At 30 min post-treatment, the mice groups treated with the MS-3 fraction showed only 0.99 °C (*p* < 0.001) and 1.04 °C (*p* < 0.01) reduction in their rectal temperatures at 250- and 500-mg/kg doses, respectively. At 120 min post-treatment, however, the mice groups treated with the MS-3 fraction showed a statistically significant reduction in their rectal temperatures (*p* < 0.001) when compared to the positive control indomethacin by 1.7 °C and 2.2 °C at 250- and 500-mg/kg doses, respectively (Table 6).

### 2.6. Molecular Docking

Docking poses showed that the major compounds identified in the MS-3 fraction of *M. sinaica* were able to fit properly in the protein-binding pockets, affording diverse interactions with the amino acid residues and demonstrating appreciable free-binding energies reflected by minimum scoring function values compared to the reference ligands co-crystalized with the target proteins, as shown in Table 7.

The endocannabinoid system is widely involved in modulating the neurotransmission, pain and inflammation responses and comprises the cannabinoid receptors 1 and 2 (CB1 and CB2), the endocannabinoid ligands and the enzymes associated with the synthesis and degradation of these ligands [19]. Cannabinoid receptor agonists have various pharmacological effects, including analgesia, anti-inflammatory, antiepileptic, neuroprotective, hypnotic and immunomodulatory effects [20].

Recently, several molecules from various phytochemical classes showed good affinity to cannabinoid receptors and/or inhibitory potential towards endocannabinoid-degrading enzymes such as fatty acid amide hydrolase (FAAH) [21]. Polyphenols, for instance, are widely renowned with their analgesic and anti-inflammatory properties [22]. Such effects could plausibly be due to targeting the endocannabinoid system. Some polyphenols demonstrated an affinity to the human cannabinoid receptor CB1 in radioligand assays such as delphinidin (Ki: 21.3 µM) and cyanidin (Ki: 16.2 µM), whereas similar binding affinities for CB2 receptors were demonstrated also by delphinidin (Ki: 34.3 µM), cyanidin (Ki: 33.5 µM) and peonidin (Ki: 46.4 µM) [23]. Some catechins such as epigallocatechin, epigallocatechin-3-*O*-gallate and epicatechin-3-*O*-gallate were reported to modulate CB1 receptors with Ki values of 35.7 µM, 33.6 µM and 47.3 µM, respectively [24]. The flavanone glycosides miconioside B and C showed weak affinity to CB2 receptors in radioligand-binding studies [25]. In an in vitro biochemical assay, some commonly occurring flavonoids were able to inhibit the FAAH enzyme involved in the hydrolysis of the endogenous cannabinoids, where kaempferol was shown to be the most potent inhibitor, with a Ki value of 5 µM [26].

**Figure 3:**
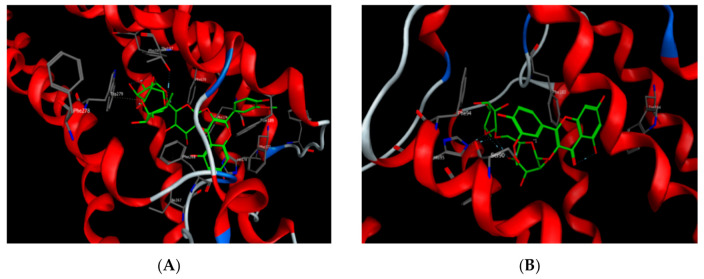
Isorhamnetin-3-*O*-(2-glucosyl) rutinoside docked onto the CB1-binding site (**A**) and isorhamnetin 3-*O*-β-glucopyranoside-7-*O*-α-rhamnopyranoside docked onto the CB2-binding site (**B**).

Most of the polyphenols identified in the MS-3 fraction of *M. sinaica* revealed good binding when they were docked to CB1 and CB2-binding sites with appreciable binding energy, as shown in Table 7. Isorhamnetin-3-*O*-(2-glucosyl) rutinoside showed the lowest binding energy to CB1 receptors, with a scoring function as low as −24.66 kcal/mol. Isorhamnetin 3-*O*-β-glucopyranoside-7-*O*-α-rhamnopyranoside, on the other hand, showed the lowest binding energy (−20.10 kcal/mol) towards the CB2 receptors (Figure 3).

**Figure 4:**
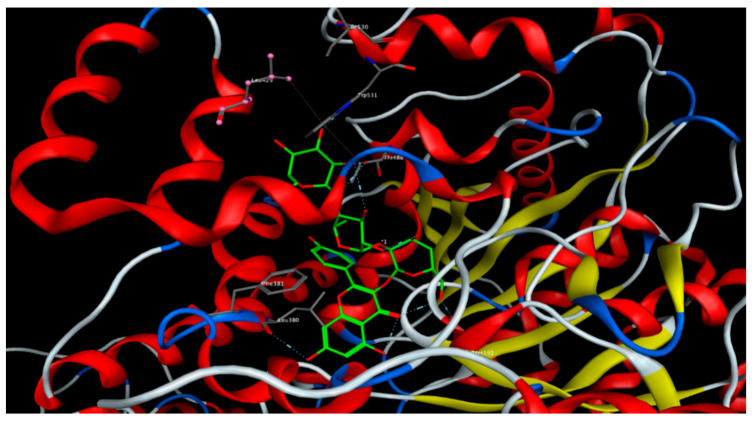
Kaempferol-3-*O*-β-(2″-*O*-glucosyl)-rutinoside docked onto the binding site of the fatty acid amide hydrolase (FAAH) enzyme.

As for the FAAH enzyme, all docked compounds showed appreciable binding energy compared to the reference inhibitor. Kaempferol-3-*O*-β-(2″-*O*-glucosyl)-rutinoside and isorhamnetin-3-O-(2-glucosyl) rutinoside showed the lowest binding energies, with scoring function values of –27.03 and –27.11 kcal/mol, respectively (Figure 4). The free aglycons quercetin, kaempferol and isorhamnetin were also docked to all the studied proteins. Quercetin showed the lowest binding energy to the CB1 and CB2 receptors and FAAH enzyme (Table 7).

The inhibition of prostaglandin biosynthesis is one of the major mechanisms involved in the anti-inflammatory activity of nonsteroidal anti-inflammatory drugs (NSAIDs) [27]. For the sake of discovering safer natural alternatives of NSAIDs, further studies are still in demand to identify novel bioactive constituents in different plants extracts, evaluate their anti-inflammatory potentials and clarify their mechanisms of action [28]. In these regards, the polyphenols identified in the *M. sinaica* (MS-3) fraction were docked to the cyclooxygenases (COX-1 and COX-2), which are two key enzymes in inflammation signaling, as they catalyze the very first step in prostaglandins synthesis from the precursor arachidonic acid [29]. The docked compounds were able to bind to the COX-1 and COX-2-binding pockets and afforded similar amino acid interactions compared to the co-crystallized ligands. As shown in Table 7, the quercetin derivatives did not show significant selectivity towards any of the two enzymes; however, the isorhamnetin and kaempferol derivatives showed to be more selective to COX-2. Isorhamnetin 3-*O*-β-glucopyranoside-7-*O*-α-rhamnopyranoside showed scoring function values of −21.69 and −27.94 kcal/mol for COX-1 and COX-2, respectively, whereas kaempferol-3-*O*-β-(2″-*O*-glucosyl)-rutinoside showed scoring function values of −23.04 and −27.49 kcal/mol for COX-1 and COX-2, respectively.

## 3. Materials and Methods

### 3.1. Chemicals and Solvents

Aluminum chloride was purchased from Sigma-Aldrich (Schnelldorf, Germany), Folin-Ciocalteu reagent from Loba-Chemie (Mumbai, India) and quercetin and gallic acids from Sigma Chemical Co. (St. Louis, MO, USA). Acetic acid, carrageenan and oxyphenbutazone from Sigma-Aldrich (St. Louis, MO, USA) and indomethacin from Kahira Pharmaceuticals Co. (Cairo, Egypt). All other chemicals were of commercially available analytical grade chemicals and solvents.

### 3.2. Plant Material and Extraction

The aerial parts of *Moricandia sinaica* Boiss. were collected from Wadi Hfr Al Batin, Saudi Arabia in March 2016. After the taxonomical identification, the specimen was deposited at the herbarium of the College of Pharmacy, King Saud University, Riyadh, Saudi under the code number SY284. The collected plant parts were washed with distilled water, dried in shade and ground to a fine powder. The powdered material (750 g) was extracted three times with 80% (*v/v*) aqueous methanol (MeOH) (3 × 2 L) at room temperature. A part of the combined extract was evaporated under reduced pressure by a vacuum rotary evaporator (Rotavapor EL-130, Vacuum Pump V-700, Vacuum Control V-850 Buchi AG, Flawil, Switzerland) until dryness to obtain MS-1 (33 g). The rest of the extract was concentrated and suspended in 120-mL distilled water, sonicated (30 min), defatted with hexane and then extracted with butanol. The butanol and the aqueous fraction were concentrated until dryness to give MS-2 (43 g) and MS-3 (58 g), respectively.

### 3.3. Preliminary Qualitative Analysis of the M. sianica Fractions

Flavonoids and phenolics were determined qualitatively according to Shinoda’s [30] and the ferric chloride method [31], respectively. Fatty acids, alkaloids, terpenes and saponins were also screened [32].

### 3.4. HPLC-PDA-MS/MS

The aqueous fraction (MS-3) was subjected to high-performance liquid chromatography-mass spectrometry (HPLC-PDA-MS/MS). Thermofinigan (Thermo Electron Corporation, Waltham, MA, USA) coupled with an LCQ-Duo ion trap mass spectrometer with an electrospray ionization (ESI) source (ThermoQuest, Austin, TX, USA) was utilized. The separation was achieved using a discovery HS FS column (4.6 × 150 mm, 5 μm; Sigma-Aldrich Co, Steinheim, Germany). A gradient of water and acetonitrile (ACN) (0.1% formic acid each) was applied starting with 5% ACN that was increased to 30% over 60 min and a flow rate of 1 mL/min with a 1:1 split before the ESI source. The MS operated in the negative mode with a capillary voltage of −10 V, a source temperature of 200 °C and high purity nitrogen as a sheath and auxiliary gas at a flow rate of 80 and 40 (arbitrary units), respectively. Collision energy of 35% was used in MS/MS fragmentation. The ions were detected in a full scan mode and mass range of 50–2000 *m/z* [33].

### 3.5. Classical Molecular Networking Workflow Description

Three main steps were followed through the design of the molecular network: firstly, the conversion of the mass file into a mgf extension using the MS program. Secondly, uploading the prepared mgf file to the GNPS online platform (http://gnps.ucsd.edu). Then, the data should be filtered by taking out all fragment ions within ±17 Da of the precursor *m/z* and choosing only the “top 6 fragment ions in the ±50 Da window throughout the spectrum, precursor ion mass tolerance was set to 2.0 Da and a MS/MS fragment ion tolerance of 0.5 Da, and edges were filtered to have a cos score ˃0.7 and more than 6 matched peaks”. Finally, the molecular networking workflow output file was visualized using Cytoscape 3.6.1 software (https://cytoscape.org/) [34].

### 3.6. Molecular Modeling

Major compounds identified in the *M. sinaica* most bioactive fraction (MS-3) were docked to some target proteins associated with analgesic and anti-inflammatory activities, namely the cannabinoid receptors CB1 and CB2, fatty acid amide hydrolase (FAAH), and cyclooxygenase COX-1 and COX-2 enzymes. The builder tool of MOE (molecular operating environment) software, 2013.08, Chemical Computing Group Inc.; Montreal, QC, Canada, H3A 2R7, 2016 was used to draw the compounds’ structures. The force field mmff94x was utilized to identify the energy-minimized conformers, which were then assigned their ionization state by the molecule wash tool of the software. The Protein Data Bank (www.pdb.org) was accessed to download the crystal structures of CB1 (ID, 5XRA), CB2 (ID, 5ZTY), COX-1 (ID, 2OYE), COX-2 (ID, 3LN1) and FAAH (ID, 3QJ9). The proteins were then assigned the geometry and protonation state. Docking was performed by adopting the triangle matcher placement method, and the scoring function London dG was used to estimate the binding free energy for the docking poses.

### 3.7. Total Phenolic Content (TPC) and Total Flavonoid Content (TFC)

The Folin-Ciocalteu method using gallic acid as a standard was used to determine the TPC of the *M. sinaica* fractions, as described before [35]. Total phenols were expressed as gallic acid equivalents (GAE) mg/g extract (dry weight, dw), using a calibration curve of a freshly prepared gallic acid solution. TFC was quantified by the aluminum chloride colorimetric method, as reported by [36,37]. The results were expressed as mg quercetin equivalents (QE) mg/g extract (dw).

### 3.8. Biological Experiments

#### 3.8.1. Animals

Albino rats aged 10–12 weeks and weighing 200 ± 20 g and Wistar mice weighing 25 ± 5 g were obtained from the Experimental Animal Care Center of the College of Pharmacy, King Saud University, Riyadh, Saudi Arabia Dec. 2017. The animals were acclimatized to our laboratory environment for seven days before running the biological experiments. They were housed in colony polypropylene cages (6 rats per cage) with regular light/dark cycles at a temperature of 25 ± 2 °C and allowed free access to standard food and water. In all experiments, the animals were divided into six groups, six animals each, receiving the different fractions. The positive control group received a reported reference drug according to the type of the biological experiment carried out. The negative control group received saline only. The study was approved (clearance no. CBR-4538) by the Research Ethics Committee of Experimental Animal Care Society, Faculty of Pharmacy, King Saud University, Riyadh, Saudi Arabia.

#### 3.8.2. Analgesic Activity

##### Hot Plate Test

The hot plate test was carried out as described by [38]. Briefly, rats in each group were placed gently on a hot plate maintained at 55 ± 5.5 °C. The reaction time was regarded as the interval extending from the instant the animal was on the hot plate until the moment the animal licked its forefeet or jumped off. The reaction time was measured 10 min before the oral administration of the fractions or the positive control and at 60, 90 and 120 min post-treatment. The different fractions were tested at 250 mg/kg and 500 mg/kg. Indomethacin (4 mg/Kg) was used as a positive control.

##### Acetic Acid-Induced Writhing in Mice

Acetic acid-induced writhing test according to the [39] model in mice was used to assess the peripheral analgesic effects of *M. sinaica* fractions. Writhing was induced in mice by the intraperitoneal administration of 0.1 mL of 1% acetic acid 30 min after the oral administration of the different treatments. The number of writhes was counted for 20 min after acetic acid injection, and the writhing percentage inhibition was calculated as per the following equation:

% inhibition = [{Average number of writhes (control) − Average number of writhes (test)}/Average number of writhes (control)] × 100.

##### Tail Flick Method

Acute nociception was induced using the tail flick apparatus (Tail Flick Apparatus, Harvard Apparatus, Holliston, MA, USA) following the method of [40]. Briefly, each rat was placed in a restrainer, and the baseline reaction time was measured 2 min before the treatment by focusing on an intensity-controlled beam of light on the distal one-third portion of the animal tail.

The tips of the animals were individually placed on the radiant heat source, and the tail flick response reaction time was recorded before and at 60, 90 and 120 min after the intraperitoneal administration of the different treatments. The fractions were tested at 250-mg/kg and 500-mg/kg doses.

#### 3.8.3. Anti-Inflammatory Activity in Carrageenan-Induced Paw Edema

Pedal inflammation in albino rats was introduced according to the method described by [41], in which 0.05 mL of 1% carrageenan sodium salt (BDH) was injected into the right hind paw of the animal just under the plant araponeurosis. The test groups were treated orally with the different fractions (250 mg/kg and 500 mg/kg) one hour before carrageenan injection. At the same time, the untreated control group received 5 mL/kg of normal saline, and the positive control group received 100 mg/kg of oxyphenbutazone aqueous solution. The measurements of the paw volumes were done by the displacement technique using a plethysmometer (Apelex, Massy, France) immediately after and at 2 and 3 h post-carrageenan injection. The percentage inhibition was calculated according to the formula:

100[1 − (a − x)/(b − y)]

where “y” and “b” are the mean paw volume of the control rats before and after carrageen injection, respectively. “x” and “a” are the mean paw volume of the treated rats before and after carrageenan injection, respectively.

#### 3.8.4. Antipyretic Activity

The antipyretic activity was evaluated using Brewer’s yeast-induced pyrexia in mice. The initial rectal temperature for each mouse was measured using a lubricated digital thermometer. Hyperthermia was induced in mice by the subcutaneous injection of a 20% aqueous suspension of Brewer’s yeast (20 mL/kg) below the nape of the neck [42]. The animals were fasted for 24 h, where they were allowed access to water only. Rectal temperature was measured again after 24 h. Only the mice that showed higher temperatures by 0.5 °C or more were included in the study. The feverish animals in the test groups received orally the different fractions of *M. sinaica* at 250- and 500-mg/kg dose levels, while those in the positive control group received 4-mg/kg oral dose of indomethacin. The rectal temperature of all animals was then measured at 60, 90 and 120 min post-treatment.

### 3.9. Statistical Analysis

Results were expressed as mean ± standard error of the mean of triplicate experiments. Data were analyzed using one-way analysis of variance (ANOVA), followed by Dunnett’s *t*-test. Results were considered significant at **p < 0.05. For total phenolic and flavonoids content results, the data were expressed as mean ± standard deviation of triplicate experiments. The statistical analysis was performed using SAS software ver. 16 (SAS Institute Inc., Cary, NC, USA). Differences among groups were evaluated by one-way analysis of variance (ANOVA), followed by Duncan’s multiple range tests. Linear regression was performed for calibration curves and for the calculations of percentage inhibition. GraphPad Prism 6.0 (Inc. La Jolla, CA, USA) software was used for data analysis.

## 4. Conclusions

In the current study, the aqueous fraction from *M. sinaica* (MS-3) exhibited potent analgesic, antipyretic and anti-inflammatory effects in the experimental animals, which are in agreement with the traditional use of *Moricandia* species in the treatment of different inflammatory and pain-associated diseases. The pharmacological properties of the fractions were comparable to those of NSAIDs, which are renowned of their antipyretic, analgesic and anti-inflammatory activities. Twenty-four secondary metabolites were identified in the MS-3 fraction using HPLC-MS/MS. The compounds included flavonoids, phenolic acids and glucosinolates. Molecular docking results revealed that polyphenols identified in the current work provide good lead compounds for the development of new safer analgesics and NSAID alternatives of natural origin, most probably lacking the adverse reactions associated with the current drugs in the medicinal market. However, further studies are required to isolate the individual compounds from the most promising bioactive fraction and explore their mechanism of action at all the levels of pain and inflammatory signaling pathways.

## Figures and Tables

**Table 1:** Secondary metabolites from the bioactive fraction (MS-3) of *Moricandia. sinaica.*

No.	t_R_	(M − H)^−^	MS/MS	Identified Secondary Metabolites	References
1	0.92	317	225, 165, 125	Galloyl phloretic acid	
2	2.66	341	179	Caffeoyl glucose	[11]
3	3.17	372	292, 259, 194, 163	Gluconapin	[16]
4	3.53	133	114.9, 115, 87, 71	Malic acid	[12]
5	5.55	385	223, 205	Sinapic acid 3-*O*-glucoside	[13]
6	7.11	163	147, 119, 106, 72	*p*-Coumaric acid	[14]
7	9.08	263	245, 179	Caffeic acid derivative	
8	11.12	355	338, 309, 193	Methyl-4-*O-β*-D-glucopyranosyl-caffeate	[15]
9	14.20	203	186, 159, 143, 116	Tryptophan	
10	17.94	771	301, 447, 609, 625	Quercetin 3,4‘-di-O-*β*-d-glucopyranoside-7-O-α-l-rhamnopyranoside ^#^	[18]
11	19.06	755	285, 447, 609	Kaempferol-3-*O*-*β*-(2″-*O*-galactosyl)-rutinoside	
12	20.15	755	285, 447, 609	Kaempferol-3-*O*-*β*-(2″-*O*-glucosyl)-rutinoside *	[15]
13	21.13	785	315, 461, 623, 639	Isorhamnetin-3-*O*-(2-glucosyl) rutinoside	
14	21.19	785	315, 461, 623, 639	Isorhamnetin-3-*O*-(2-glucosyl) rutinoside	
15	22.04	947	315, 609, 771, 801	Isorhamnetin-3-*O*-rutinoside-7-*O*-diglucoside	
16	24.02	977	315, 639, 771, 785, 831	Isorhamnetin-3-*O*-feruloyl glucoside-7-*O*-diglucoside	
17	24.71	609	179, 301, 447, 463	Quercetin-3-*O*-*β*-glucosyl-7-*O*-α-rhamnoside *	[15]
18	25.41	771	301, 447, 625	Quercetin-3-O-*β*-sophoroside-7-O-*α*- rhamnoside	[15]
19	28.47	785	315, 461, 639	Isorhamnetin-3-*O*-(2-glucosyl) rutinoside	
20	29.69	623	315, 461, 477	Isorhamnetin 3-*O*-*β*-glucopyranoside-7-*O-α*-rhamnopyranoside	
21	31.33	977	315, 639, 771, 785, 831	Isorhamnetin-3-*O*-feruloyl glucoside-7-*O*-diglucoside	
22	32.34	947	315, 639, 785, 801	Isorhamnetin 7-*O*-dicaffeoyl-3-*O*-rutinoside	
23	36.54	755	285, 593, 431	Kaempferol-3-*O*-*β*-sophoroside-7-*O*-α-rhamnoside	[15]
24	37.71	593	285, 431, 447	Kaempferol-3-*O*-*β*-glucosyl-7-*O*-α -rhamnoside	[15]

*^#^* Previously isolated in *Moricandia arvensis* [18]. * Previously identified in *M. arvensis* [15]. t_R_: retention time and MS: mass fragments.

**Table 2:** Analgesic effect of *M. sinaica* fractions on the hot plate reaction time in mice.

Treatment	Dose (mg/kg)	Reaction Time (Seconds) Pretreatment	Reaction Time (Seconds) Post-Treatment		
			30 min	60 min	120 min
MS-1	250	8.00 ± 0.36	7.00 ± 0.44	8.16 ± 0.30	8.16 ± 0.30
MS-1	500	7.50 ± 0.42	8.16 ± 0.47	8.50 ± 0.42	8.66 ± 0.42
MS-2	250	7.66 ± 0.49	8.83 ± 0.30	9.00 ± 0.36	9.50 ± 0.42 *
MS-2	500	6.83 ± 0.30	10.00± 0.36 ***	11.00 ± 0.51 ***	10.33 ± 0.49 ***
MS-3	250	7.33 ± 0.42	9.66 ± 0.49 **	10.66 ± 0.49 ***	10.83 ± 0.30 ***
MS-3	500	7.16 ± 0.30	10.50 ± 0.42 ***	11.83 ± 0.30 ***	11.66 ± 0.42 ***
Indomethacin	4	7.33 ± 0.42	12.16 ± 0.47 ***	14.00 ± 0.36 ***	14.50 ± 0.42 ***

All values represent mean ± SEM, *n* = 6; * *p* < 0.05, ** *p* < 0.01 and *** *p* < 0.001 by one-way ANOVA.

**Table 3:** Analgesic effects of *M. sinaica* fractions on acetic acid–induced writhing in mice.

Treatments	Dose (mg/kg)	Number of Writhing in 20 min.	% Inhibition
Control (Acetic acid)	0.1 mL of 20%	36.83 ± 1.53	-
MS-1	250	34.50 ± 1.17	6.33
MS-1	500	36.66 ± 2.21 *	16.74
MS-2	250	29.00 ± 1.06 **	21.26
MS-2	500	21.66 ± 0.71 ***	41.17
MS-3	250	20.50 ± 1.17 ***	44.34
MS-3	500	14.16 ± 0.60 ***	61.53
Indomethacin	4	6.83 ± 0.60 ***	81.44

All values represent mean ± SEM, *n* = 6; * *p* < 0.05, ** *p* < 0.01 and *** *p* < 0.001 by ANOVA, followed by Dunnett’s multiple comparisons test.

**Table 4:** Analgesic effects of *M. sinaica* fractions on the tail flick method in mice.

Treatment	Dose (mg/kg)	Pre-Drug	Response Time Duration (Seconds) Post-Drug		
			30 m	60 m	120 m
MS-1	250	3.66 ± 0.33	4.16 ± 0.30	3.83 ± 0.30	4.00 ± 0.36
MS-1	500	4.16 ± 0.30	4.83 ± 0.30	5.50 ± 0.22 **	4.83 ± 0.40
MS-2	250	3.83 ± 0.30	5.83 ± 0.30 ***	6.33 ± 0.33 ***	6.33 ± 0.33 ***
MS-2	500	4.66 ± 0.21	6.83 ± 0.30 ***	7.16 ± 0.40 ***	7.50 ± 0.56 ***
MS-3	250	4.50 ± 0.22	6.50 ± 0.34 ***	7.33 ± 0.33 ***	7.5 ± 0.42 ***
MS-3	500	5.16 ± 0.30	7.83 ± 0.30 ***	9.16 ± 0.30 ***	8.66 ± 0.49 ***
Indomethacin	4	4.50 ± 0.42	9.33 ± 0.55 ***	10.50 ± 0.42 ***	11.00 ± 0.36 ***

All values represent mean ± SEM, *n* = 6; ** *p* < 0.01 and *** *p* < 0.001 by ANOVA, followed by Dunnett’s multiple comparisons test.

**Table 5:** Effects of *M.* sinaica fractions on carrageenan-induced paw edema in Albino rats.

Extract	Dose	Before Carrageenan	Carrageenan	Change	Inhibition
	(mg/kg)		After 3 h		(%)
Carrageenan	0.05 mL of 1%	0.98 ± 0.03	1.57 ± 0.02	0.58 ± 0.01	-
MS-1	250	0.98 ± 0.04	1.54 ± 0.02	0.56 ± 0.03	3.70
MS-1	500	1.00 ± 0.02	1.54 ± 0.01	0.53 ± 0.01	8.54
MS-2	250	1.03 ± 0.02	1.43 ± 0.01	0.40± 0.01 ***	31.33
MS-2	500	1.01 ± 0.03	1.30 ± 0.02	0.29 ± 0.20 ***	50.42
MS-3	250	0.95 ± 0.04	1.27 ± 0.04	0.32 ± 0.01 ***	45.00
MS-3	500	0.91 ± 0.02	1.19 ± 0.01	0.27 ± 0.01 ***	52.42
Oxyphenbutazone	100	1.00 ± 0.04	1.19 ± 0.04	0.19 ± 0.01 ***	66.66

All values represent mean ± SEM, *n* = 6; *** *p* < 0.001 by ANOVA, followed by Dunnett’s multiple comparisons test.

**Table 6:** Effects of *M. sinaica* and fractions on yeast-induced hyperthermia in mice.

Treatment	Dose (mg/kg)	Normal Rectal Temperature	Rectal Temperature after Yeast Administration	Rectal Temperature °C Post-Treatment		
				30 min	60 min	120 min
MS-1	250	35.3 ± 0.09	38.46 ± 0.16 ***	38.15 ± 0.12	38.18 ± 0.15	38.10 ± 0.12 *
MS-1	500	35.31 ± 0.10	38.21 ± 0.12 ***	37.88 ± 0.07 *	37.88 ± 0.09	37.98 ± 0.14
MS-2	250	35.28 ± 0.11	38.31 ± 0.17 ***	37.88 ± 0.14	37.43 ± 0.16 **	37.50 ± 0.15 **
MS-2	500	35.46 ± 0.15	38.56 ± 0.16 ***	37.55 ± 0.17 **	37.25 ± 0.21 ***	37.26 ± 0.11 ***
MS-3	250	35.33 ± 0.10	38.60 ± 0.15 ***	37.61 ± 0.13 ***	37.05 ± 0.09 ***	36.86 ± 0.14 ***
MS-3	500	35.20 ± 0.12	38.50 ± 0.15 ***	37.46 ± 0.20 **	36.56 ± 0.11 ***	36.35 ± 0.08 ***
Indomethacin	4	35.51 ± 0.10	38.86 ± 0.10 ***	36.51 ± 0.19 ***	36.08 ± 0.08 ***	35.70 ± 0.10 ***

All values represent mean ± SEM, *n* = 6; * *p* < 0.05, ** *p* < 0.01 and *** *p* < 0.001 by ANOVA, followed by Dunnett’s multiple comparisons test.

**Table 7:** Scoring functions of the docking poses of the major compounds from the MS-3 fraction of *M. sinaica* docked to the cannabinoid receptors CB1 and CB2 and fatty acid amide hydrolase (FAAH) and cyclooxygenase (COX-1 and COX-2) enzymes.

Compound	Scoring Function (kcal/mol)				
	CB1	CB2	FAAH	COX-1	COX-2
Quercetin 3,4‘-di-*O*-*β*-d-glucopyranoside-7-O-α-l-rhamnopyranoside	−23.51	Failed	−23.52	−25.43	−23.75
Kaempferol-3-*O*-*β*-(2″-*O*-glucosyl)-rutinoside	−21.46	Failed	−27.03	−23.04	−27.49
Isorhamnetin-3-*O*-(2-glucosyl) rutinoside	−24.66	Failed	−27.11	−21.85	−27.23
Quercetin-3-*O*-β-glucosyl-7-*O*-α-rhamnoside	−22.58	−17.66	−26.20	−24.23	−23.94
Isorhamnetin 3-*O*-β-glucopyranoside-7-*O*-α-rhamnopyranoside	−22.45	−20.10	−25.19	−21.69	−27.94
Quercetin	−12.02	−13.80	−16.04	−16.18	−16.07
Kaempferol	−11.20	−12.76	−13.51	−14.90	−13.05
Isorhamnetin	−12.20	−12.06	−15.31	−14.40	−15.12
Diclofenac				−10.36	−12.30
AM11542 (CB1 agonist)	−14.22				
HU308 (CB2 agonist)		−13.10			
Ketobenzimidazole derivative (FAAH inhibitor)			−14.18		

## Abbreviations

EC_50_: Half maximal effective concentration; DPPH: 2 2 diphenyl 1 picrylhydrazyl radical; HPLC-PDA-MS: High Performance Liquid Chromatography -Photodiode Array Detector-Mass Spectrometry; NSAIDs: Non-steroidal anti-inflammatory drugs.
